# QUALITY OF LIFE AND PERCEIVED NEEDS AMONG OLDER ADULTS RECEIVING LONG-TERM SERVICES AND SUPPORTS

**DOI:** 10.1093/geroni/igz038.2329

**Published:** 2019-11-08

**Authors:** Eleanor Rivera, Karen Hirschman, Mary Naylor

**Affiliations:** University of Pennsylvania, Philadelphia, Pennsylvania, United States

## Abstract

Long term services and supports (LTSS) are vital for older adults with physical and cognitive disabilities. LTSS can be provided in settings such as nursing homes, assisted living, or via community-based services. The aim of this study is to describe the perceived needs for older adults new to LTSS, examine whether those needs are met in the first three months of LTSS, and determine the relationship with quality of life (QoL). This secondary analysis included data from 470 older adults new to LTSS (average age: 81, 71% female, 51% white, 35% black, 20% Hispanic.) The main outcome of QoL was measured using a single item (“How would you rate your overall quality of life at the present time?”). Perceived needs included supportive equipment devices, transportation, physical therapy, and social activities. Analyses at baseline and three months included t-tests, ANOVAs and simple regression modeling. LTSS recipient reported needs at baseline were: 29% supportive equipment, 31% transportation, 20% physical therapy, and 25% social activities. Those who reported needs at baseline had a lower QoL than those who reported no needs (for all). At three months reported needs decreased by an average of 6% (range: 3%-10%). QoL ratings were associated with changes in physical therapy and social activities needs at three months. The implications of these results related to LTSS recipients’ QoL in the first three months of services, with emphasis on physical therapy and social activities needs, is an opportunity to be more person-centered in delivery of care.

